# Risk Prediction for Sudden Cardiac Death in the General Population: A Systematic Review and Meta-Analysis

**DOI:** 10.3389/ijph.2024.1606913

**Published:** 2024-03-20

**Authors:** Yue Li, Zhengkun Liu, Tao Liu, Ji Li, Zihan Mei, Haojun Fan, Chunxia Cao

**Affiliations:** ^1^ College of Management and Economics, Tianjin University, Tianjin, China; ^2^ Institute of Disaster and Emergency Medicine, Tianjin University, Tianjin, China

**Keywords:** risk prediction, risk factors, meta-analysis, predict factors, sudden cardiac death

## Abstract

**Objective:** Identification of SCD risk is important in the general population from a public health perspective. The objective is to summarize and appraise the available prediction models for the risk of SCD among the general population.

**Methods:** Data were obtained searching six electronic databases and reporting prediction models of SCD risk in the general population. Studies with duplicate cohorts and missing information were excluded from the meta-analysis.

**Results:** Out of 8,407 studies identified, fifteen studies were included in the systematic review, while five studies were included in the meta-analysis. The Cox proportional hazards model was used in thirteen studies (96.67%). Study locations were limited to Europe and the United States. Our pooled meta-analyses included four predictors: diabetes mellitus (ES = 2.69, 95%CI: 1.93, 3.76), QRS duration (ES = 1.16, 95%CI: 1.06, 1.26), spatial QRS-T angle (ES = 1.46, 95%CI: 1.27, 1.69) and factional shortening (ES = 1.37, 95%CI: 1.15, 1.64).

**Conclusion:** Risk prediction model may be useful as an adjunct for risk stratification strategies for SCD in the general population. Further studies among people except for white participants and more accessible factors are necessary to explore.

## Introduction

Sudden cardiac arrest (SCD) is a serious complication of atherosclerosis. SCD is defined as an abrupt and unexpected loss of cardiovascular function resulting in circulatory collapse and death [1]. Generally, sudden cardiac arrest (SCA) has two distinct outcomes: SCD or aborted SCD (i.e., sudden cardiac arrest survivors) [2]. SCA mortality is approximately 90% and significant functional and/or cognitive disabilities often persist among those who survive [3].

SCD accounts for over 4-5 million deaths a year [4], and is the leading cause of death, accounting for 10%–20% of deaths globally [5]. In the general population, the relative risk of SCD is significantly lower than in patients with cardiac disease [6]. Despite this, the absolute number of people who suffer from SCA/SCD is greater than those with cardiac disease due to the sheer size of the population at risk [7].

Among individuals in the general population, up to 50% of SCD is their first manifestation of cardiac disease [8, 9]. Symptoms of SCD typically appear soon after the onset of the first symptoms, leaving little or no time for effective medical interventions [10]. Therefore, identifying individuals at risk for SCD is important from a clinical and public health perspective. The left ventricular ejection fraction (LVEF), which is currently used to identify candidates for primary prevention implantable cardioverter-defibrillators (ICDs), has significant limitation [1]. There have been numerous proposals for new tools for risk stratification to date. However, systematic reviews or meta-analyses inadequately explored predictors of SCD risk. To our knowledge, only one systematic review about risk prediction models for SCD, which included articles until 2019 [11]. Our objective was to summarize and appraise available prediction models for SCD risk in the current systematic review and meta-analysis.

## Methods

The systematic review and meta-analysis were conducted in accordance with the Preferred Reporting Items for Systematic Reviews and Meta-Analysis (PRISMA) guidelines [12]. The study protocol was registered on the international prospective register of systematic reviews PROSPERO (ID number CRD42023415462).

### Literature Search Strategy

We performed an electronic literature search of PubMed, Embase, Cochrane Library (including CENTRAL, NIH registry, and CTRI), Web of Science, CINAHL and OpenGrey databases with no date restrictions. Searches were performed on 1 March 2023. Search terms were constructed by combining Medical Subject Headings (MeSH) with corresponding free words associated with the following keywords: “sudden cardiac death,” “ventricular fibrillation,” “ventricular tachycardia,” “risk assessment,” “risk prediction” etc., The search strategy was customized for each database (Supplementary Appendix SI). Animal studies and book/conference materials were excluded, and non-English articles were excluded.

### Study Selection

The study selection was performed by two authors (Y.L. and Z.H.M.) independently in accordance with the preset inclusion and exclusion criteria. Inter-rater reliability values were calculated using Cohen’s Kappa (K). Any disagreements were settled by consulting a third reviewer (C.X.C.).

Exclusion Criteria:

• The outcomes of the study were not ventricular fibrillation, ventricular tachycardia, SCA and SCD.
• Type of study: conference proceedings, editorials, reviews, meta-analysis, protocol and short reports.
• Imminent prediction of SCD risk (<1 day).
• That did not develop a prediction model.
• Only genetic status analysis or image analysis.
• The study population was not the general population (aged 18 and older).

Meta-analyses were conducted only when the identified predictors were statistically (i.e., type of effect estimates) and clinically homogeneous (e.g., similar reference groups for categorical variables). Meta-analysis exclusion criteria:

• Studies missing statistical information required for a forest plot analysis.
• When duplicate cohorts are identified using the criteria below, the study with the largest sample size is included in the meta-analysis: (i) A study cohort taken from the same registry as another study cohort. (ii) The same outcome (s) were analyzed. (iii) Overlap between study periods.

### Data Extraction

Two investigators (Y.L. and Z.H.M.) independently extracted the data from the selected studies. Third authors (C.X.C.) were consulted to resolve disagreements. The following information was recorded: the name of the first author, publication year, journal, study location, design, study type, sample size and the number of cases of development population and validate population, follow-up years, model’s specifications (i.e., intercept, type and the effect size (ES), and the corresponding 95% Confidence Intervals or CIs), and performance measures (i.e., C statistic, etc.).

### Risk of Bias Assessment

We used the Prediction Model Study Risk of Bias Assessment Tool (PROBAST) to assess bias. PROBAST was designed specifically to evaluate the risk of bias associated with a prediction model [13]. PROBAST consists of four domains: participants, predictors, outcomes, and analysis, with a total of 20 signaling questions. Based on the answers to the signaling questions, each domain was classified as low, high, or unclear risk of bias. Two review authors (Y.L. and Z.H.M.) independently scored the key studies, with discrepancies settled by C.X.C.

### Statistical Analysis

ESs included hazard ratios (HRs) and risk ratios (RRs). When two or more studies with no duplicate cohorts explored the same predictive variable in the systematic review, the HRs were pooled in the meta-analysis and RRs were considered equivalent [14, 15]. The inconsistency index (*I* ^*2*^ statistic) was used to assess heterogeneity. We considered *I* ^*2*^ of <30% to indicate low heterogeneity between studies, a value of 30%–60% to indicate moderate heterogeneity, and a value of >60% to indicate substantial heterogeneity. Substantial heterogeneity was defined as an *I* ^*2*^ value >50%. When trials were heterogeneous, the random-effects model was used to calculate the pooled HRs and 95% CI. In all other cases, fixed-effects models were used. Forest plots were used to display pooled outcomes.

In the study protocol, it was initially intended to conduct subgroup analyses to examine the effects of various confounding variables on heterogeneity within the pooled data. In addition, if studies were suspected of publication bias, we planned to conduct a sensitivity analysis after excluding outliers. Due to the fact that all pooled analyses contained two studies, this was not possible.

All statistical analyses were performed in STATA software (version 12.0, StataCorp LP, College Station, Texas, United States), and two-sided *p* values of <0.05 were regarded as indicate nominal statistical significance.

## Results

### Study Selection

**FIGURE 1:**
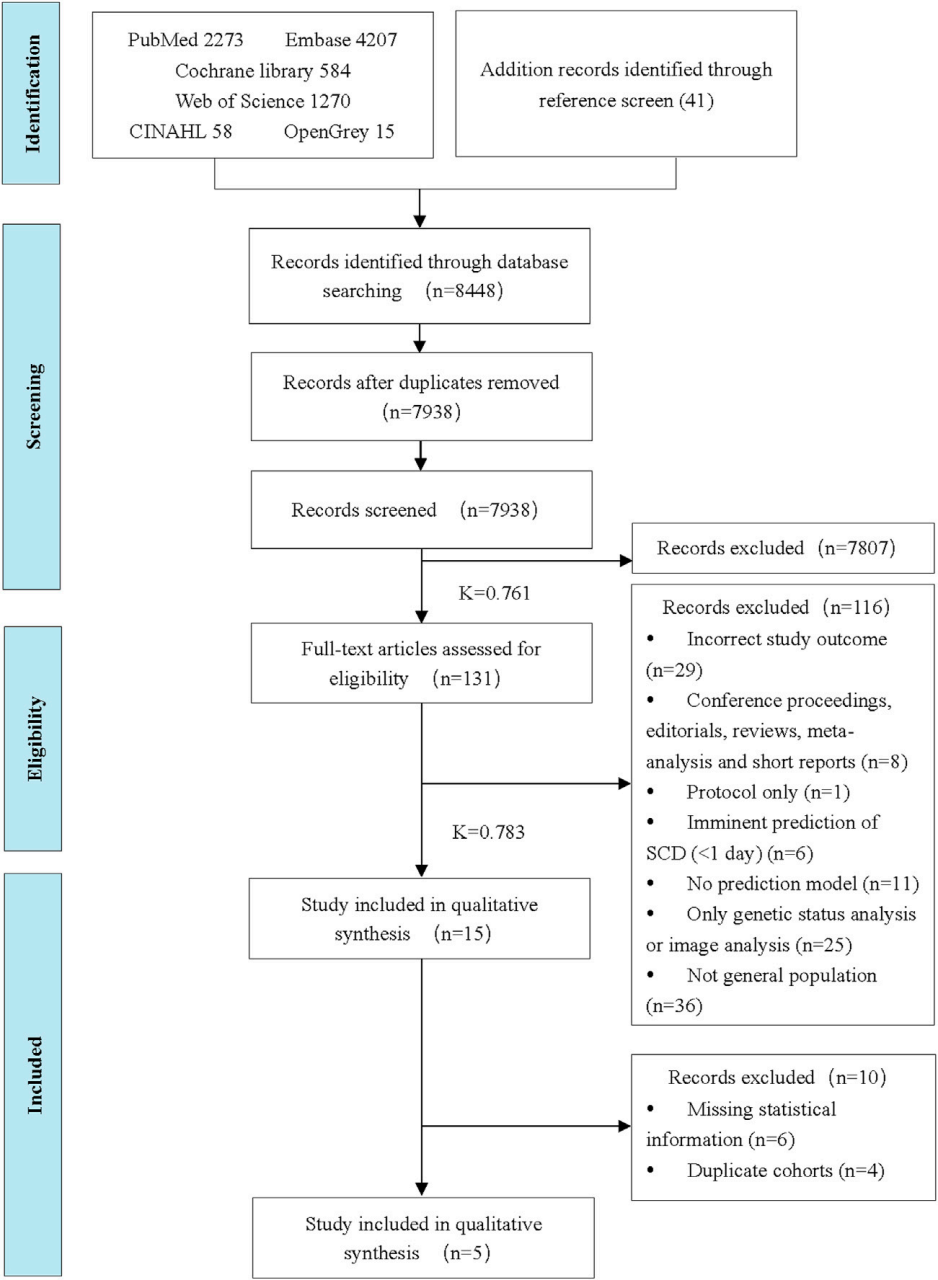
Preferred Reporting Items for Systematic Reviews and Meta-Analysis flow diagram of the study selection process (China, 2024). Note: Inter-rater reliability values were calculated using Cohen’s Kappa (K).

There were 8,448 titles and abstracts found in the literature search. Finally, fifteen studies were included in the systematic review, and five studies were eligible for the meta-analysis. A flowchart (Figure 1) illustrates the detailed selection process and reasons for exclusion.

### Characteristics of Included Studies

The key characteristics of included studies are summarized in Table 1. A systematic review of fifteen studies included 149,076 individuals (range 42–100 years old, 49.49% males) and 3,843 cases of SCD. The median follow-up was 14.10 years (range: 6.30–25.40). All studies were published between 2012 and 2023. The remaining studies were conducted in the United States (8, 53.33%), Finland (4, 26.67%), Netherlands (2, 13.33%) and China (1, 6.67%). There were fourteen cohort studies (93.33%) and 1 population-based case-control study (6.67%). Four studies mentioned no data are available in data availability statement, other studies did not mention data availability. The Definition of SCD of included studies are summarized in Supplementary Table S1.

**TABLE 1:** Characteristics of included studies (China, 2024).

First author	Publication year	Study location	Design	Study type	Development				Validate				Follow-up years^a^	Model name
					P	C	Men (%)	Age (y)	P	C	Men (%)	Age (y)		
Sudhir Kurl	2012	Finland	Cohort study	Developed model	2,049	156	100	52.7 ± 5.1	—	—	—	—	19	Cox proportional hazards model
Laukkanen Jari A	2014	Finland	Cohort study	Developed model	905	63	100	50.5 ± 6.6	—	—	—	—	20	Cox proportional hazards model
Sudhir Kurl	2015	Finland	Cohort study	Developed model	2,358	205	100	52.8 ± 5.0	—	—			20	Cox proportional hazards model
Jonathan W. Waks	2016	United States	Cohort study	Developed model	20,177	291	44	59.3 ± 10	—	—			14.1	Cox proportional hazards model
Rajat Deo	2016	United States	Cohort study	Developed and validated model	13,677	171	44	54.0 ± 6	4,207	174	39	72.0 ± 5	13.1	Cox proportional hazards model
Suma H. Konety	2016	United States	Cohort study	Developed and validated model	2,383	44	36	58.8 ± 5.7	5,366	275	42	72.9 ± 5.6	7.3	Cox proportional hazards model
Maartje N. Niemeijer	2016	Netherlands	Cohort study	Developed model	4,686	68	42	71.8 ± 7.4	—	—	—	—	6.3	Cox proportional hazards model
Takeki Suzuki	2016	United States	Cohort study	Developed model	13,070	205	33 (SCD); 66 (no SCD)	59.6 ± 5.5 (SCD); 56.9 ± 5.7 (no SCD)	—	—	—	—	11.2	Cox proportional hazards model
Aapo L. Aro	2017	United States	Population-based case-control study	Developed and validated model	1,258	522	66	65.3 ± 14.5 (case); 65.8 ± 11.5 (control)	15,792	260	55	45–65	-	Logistic regression model
Brittany M. Bogle	2018	United States	Cohort study	Developed and validated model	11,335	145	47	54.4	5,626	64	48	48.1	10	Cox proportional hazards model
Arttu holkeri	2019	Finland	Cohort study	Developed and validated model	6,830	123	45.5	51.2 ± 13.9	10,617	115	52.7	44.0 ± 8.5	24.3	Cox proportional hazards model
Leonardo Tamariz	2019	United States	Cohort study	Developed model	6,447	—	—	—	—	—	—	—	—	Cox proportional hazards model
Yun-Jiu Cheng	2021	United States	Cohort study	Developed model	14,708	706	45.6	54.3	—	—	—	—	25.4	Fine and Gray model
Anna C. van der Burgh	2022	Netherlands	Cohort study	Developed model	9,687	243	43.3	65.3 ± 9.9	—	—	—	—	8.9	Cox proportional hazards and Fine and Gray model
Yun-Yu Chen	2023	China	Cohort study	Developed model	2,105	13	47.3	≥35	—	—	—	—	15	Negative binomial regression model and Cox proportional hazard model

^a^ means median or mean of follow-up years; *P* means the number of the study population; C means the number of outcomes in the study population.

### Quality of Evidence and Risk of Bias

In assessing the overall bias and applicability of using PROBAST, twelve studies were considered high risk of bias [16–27] and three studies as low risk [28–30] (Table 2; Supplementary Figure S1). The high risk of bias was due to the model handling categorical predictors inappropriately [21–24], case-control design [22], inappropriate inclusions and exclusions of participants [16, 29], outcome [24] and overfitting optimism not being accounted for during analysis [16–21, 24–27]. One study was scored as high risk for applicability due to outcome [24], whilst two studies were scored as an unclear risk due to Participate [5, 24].

**TABLE 2:** Prediction model study risk of bias assessment tool scores of fifteen studies meeting inclusion criteria (China, 2024).

First author	Risk of bias				Applicability			Overall applicability	Risk of bias
	Participate	Predictors	Outcome	Analysis	Participant	Predictors	Outcome		
Sudhir Kurl	?	+	+	-	+	+	+	-	+
Jari A	+	+	+	-	+	+	+	-	+
Sudhir Kurl	+	+	+	-	+	+	+	-	+
Jonathan W. Waks	+	+	+	-	+	+	+	-	+
Rajat Deo	+	+	+	+	+	+	+	+	+
Suma H. Konety	+	+	+	+	+	+	+	+	+
Maartje N. Niemeijer	+	+	+	-	+	+	+	-	+
Takeki Suzuki	+	+	+	-	+	+	+	-	+
Aapo L. Aro	-	+	+	-	+	+	+	-	+
Brittany M. Bogle	+	+	+	+	+	+	+	+	+
Arttu holkeri	+	+	+	-	+	+	+	-	+
Leonardo Tamariz	?	+	+	-	?	+	-	-	-
Yun-Jiu Cheng	+	+	+	-	+	+	+	-	+
Anna C. van der Burgh	+	+	+	-	+	+	+	-	+
Yun-Yu Chen	?	+	+	-	+	+	?	-	?

Notes: “+” indicates low risk of bias/low concern for applicability, “-” high risk of bias/high applicability, and “?” unknown risk of bias/low concern for applicability.

### Analysis of the Prediction Model

In total, fifteen studies developed models to predict SCD risk, of which five studies validated the models. The Cox proportional hazards model was used in thirteen studies (96.67%), the Fine and Gray model was used in two studies (0.13%), the Logistic regression model was used in 1 study (0.07%), and the negative binomial regression model was used in 1 study (0.07%).

The predictors for SCD risk included socio-demographics, risk factors, family history, clinical history, electrocardiograph (ECG), serum measure factors, echocardiographic factors and others. The most frequent predictors were: age (14, 93.33%), diabetes mellitus (12, 80.00%), smoking (11, 73.33%), systolic blood pressure (10, 66.67%) and gender (10, 66.67%). Supplementary Figure S2 presents the frequency with which each variable was entered in the final prediction model.

**FIGURE 2:**
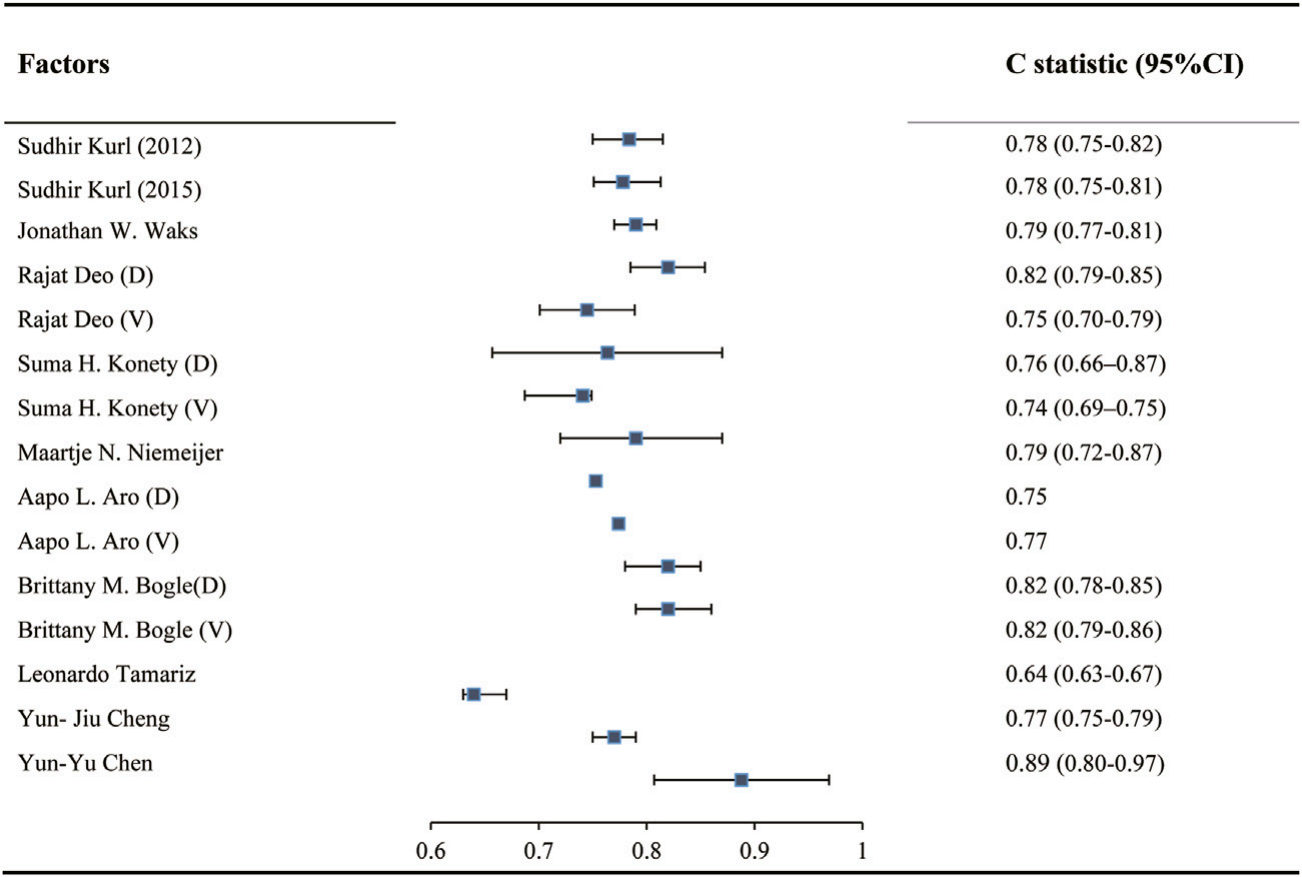
C statistic in development and validation of models predicting sudden cardiac death risk in the general population (China, 2024). Notes: “D” indicates C statistic in Developed model. “V” indicates C statistic in validated model.

In twelve studies, the C statistic was used to evaluate the model discrimination. During model development, the C statistic ranged from 0.75 to 0.90, while during model validation, the C statistic ranged from 0.74 to 0.82 (Figure 2). Calibration ofmodel was assessed using a Hosmer-Lemeshow chi-square test in two of these studies [28, 30], in which *p* values in both the derivation and validation cohort were greater than 0.05.

### Pooled Outcomes in the Meta-Analysis

**FIGURE 3:**
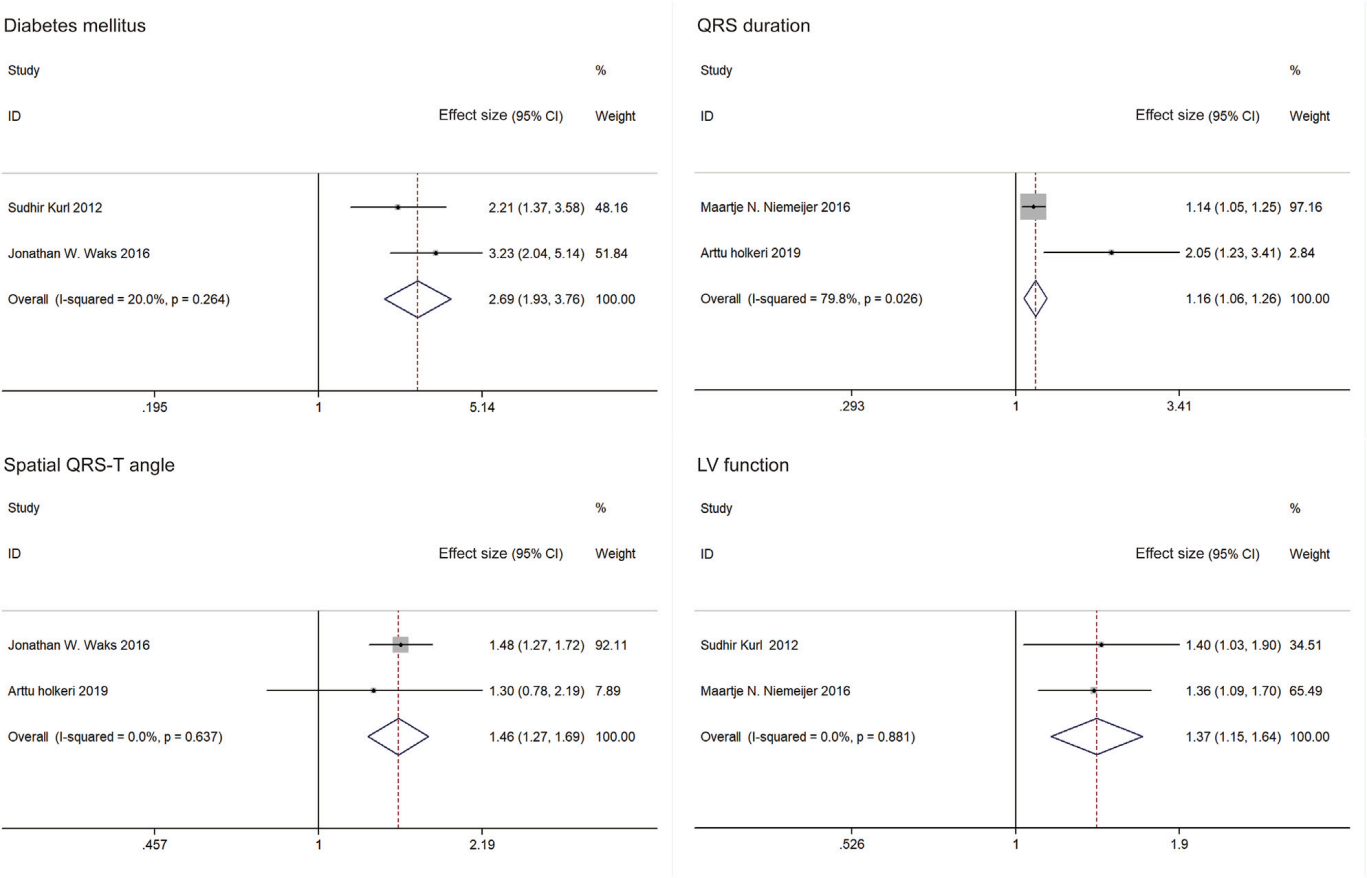
Forest plots and pooled effect estimates of predictors in models predicting sudden cardiac death risk in the general population (China, 2024). Notes: Effect size included hazard ratios (HRs) and risk ratios (RRs). Only effect sizes of study by Sudhir Kurl (2012) were RRs. Others were HRs.

The five studies in the meta-analysis included 61,409 individuals and 1,730 SCDs. The following variables predicting SCD risk in the general population had significant ESs with similar directionality across all studies in which they were significant (≥two studies): diabetes mellitus, QRS duration, spatial QRS-T angle and LV function (Figure 3). The meta-analysis included five studies that examined four predictors of SCD risk. Pooled results for the two studies demonstrate that diabetes mellitus increased SCD risk (ES = 2.69, 95%CI: 1.93, 3.76). A fixed effect estimate was used due to observed heterogeneity (*I* ^*2*^ = 20%, *p* = 0.264). QRS duration (ES = 1.16, 95%CI: 1.06, 1.26) and spatial QRS-T angle (ES = 1.46, 95%CI: 1.27, 1.69) were shown to significantly increase SCD risk. Random effects estimate of QRS duration was applied (*I* ^*2*^ = 80%, *p* = 0.026), and a fixed effect estimate of QRS duration was used (*I* ^*2*^ = 0, *p* = 0.637). Factional shortening also predicted a higher SCD risk on pooled analysis (ES = 1.37, 95%CI: 1.15, 1.64). A fixed effects estimate was used (*I* ^*2*^ = 0, *p* = 0.881). As only two studies were included in the meta-analysis of each predictor, no sensitivity analysis was performed.

## Discussion

### Key Findings

In this study, fifteen studies were included in the systematic review, of which five studies were included in the meta-analysis. Most studies were conducted in Europe and the United States. We found that the factors for predicting SCD risk included socio-demographics, risk factors, family history, clinical history, electrocardiograph (ECG), serum measure factors, echocardiographic factors, etc. In addition, diabetes mellitus, QRS duration, spatial QRS-T angle and LV function were associated with SCD risk in the meta-analysis.

SCD is a major cause of death worldwide, and more than 50% of SCDs occur in the general population. Even though there has been some progress in predicting risk of SCD in specific diseases, the greatest challenge lies in identifying the relatively small, high-risk subgroups within the large general population. The study summarized and appraised available targeted screening tools for SCD risk to identify those who may be at risk for SCD at the right time. The results provide a step toward the identification of SCD risk in the general population and can contribute to developing future strategies to prevent SCD in the community. A detailed evaluation by a cardiologist can be considered for those suspected of heart disease or risk on initial screening, to optimize the timing of interventions, and better implement evidence-based monitoring or management.

### Characteristics of Included Studies

Our results show that fifteen studies were published in the past 10 years. A study by Sudhir Kurl et al. examined the relationship between QRS duration and SCD in a population-based sample of men using Cox proportional hazards models, which was published in 2012 [16]. This period was divided into two stages based on the number of articles: Stage 1, from 2012 to 2015, was considered to be the initial period. Since 2016, stage 2 has been referred to as the development phase. Of note, 5 articles were published in 2016. Research interests in SCD risk models were prominent.

### Predictors of the Model

This finding indicates that a very high-risk group of SCD among the general population may identified using risk score for screenings. Alongside a concise history taking and simple blood test, screening tools including ECG and echocardiography can be an effective, targeted screening tool to identify those who may be at risk for SCD at the right time [31]. While, it is indicated that further studies among more accessible factors are necessary to explore.

Specifically, we found an almost 3-fold increased risk in the diabetes mellitus approximately. Epidemiological studies have shown that diabetes confers an incremental risk of SCD beyond its usual association with CAD. There was a greater risk of SCD among people with diabetes than among those without it in a meta-analysis primarily consisting of individuals over the age of 50 years (risk ratio, 2.02; 95% CI, 1.81–2.25) [32]. The prevalence of SCD among those aged 1–35 and 36–49 years was associated with approximately an eight- and a sixfold increase in diabetes, respectively [33].

ECGs are widely available, inexpensive, non-invasive tools that are well known to all physicians to predict SCD [34]. Of note, we found QRS duration and spatial QRS-T angle were statistically significant predictors. In patients with prolonged QRS duration, tachyarrhythmias tend to be more complex, more likely to degenerate, and to have a higher rate of sudden cardiac death [35]. Through the facilitation of reentrant tachyarrhythmias, the prolonged QRS with perturbed depolarization may play a direct role in SCD. An analysis of a general population by Aro et al. concluded that QRS duration ≥110 ms is a significant risk factor for SCD with a 2-fold increase in risk [36]. Based on a 10-year analysis, QRS duration was found to be a risk factor for SCD, however, a longer follow-up period failed to provide any value in predicting SCD risk [36]. Nevertheless, it remains uncertain whether QRS duration is an independent marker for SCD or merely a manifestation of more advanced cardiovascular disease.

Furthermore, the spatial and frontal QRS-T angles have been studied for decades using vectorcardiography to determine depolarization and repolarization, and this field of study has reemerged recently due to an increased risk of cardiac death and sudden cardiac death [37]. Several studies using large general populations have found that QRS-T angle is a significant risk factor for adverse cardiac events [37]. Our study yielded parallel results. There was a significant difference in 10-year risk prediction. It has been found by Henri K. Terho that QRS duration is greater than 110 ms, QRST-angle greater than 100°, left ventricular hypertrophy, and T-wave inversions are the most significant independent ECG risk markers with a 3-fold risk of developing SCD over the next 10 year [38].

On a pooled analysis, factor shortening also predicted a higher risk of SCD. The most commonly used factor for risk stratification of SCD is decreased left ventricular ejection fraction (LVEF) [39]. In current practice guidelines, an LVEF of less than 35% is a major criterion for ICD therapy [1]. However, only 20%–30% of ICD recipients in randomized clinical trials receive appropriate ICD shocks over the course of 4 years, which reduces the positive predictive value of LV dysfunction as a marker [40]. Additionally, according to population cohort studies, approximately 65% of those who suffer SCD have either normal or mildly depressed LV function (i.e., a reduced ejection fraction of 35%–50% [41, 42]. The severity of LV dysfunction alone does not provide a sufficient marker for SCD, however it could be useful when combined with other factors or as part of a multivariable risk calculation.

### Analysis of the Prediction Model

SCD has been variably defined in epidemiologic studies depending on available data, which is relevant for the interpretation of the results, The accepted and widely used definition of sudden cardiac death is “it occurred either within 1 h after the onset of an abrupt change in symptoms or within 24 h after onset of symptoms.” Outside of Europe and the United States, there is little evidence to support the prediction of SCD risk in relation to race. White participants constituted the majority of the study population. Epidemiological studies have shown that the risk of cardiac arrest varies with race [43]. In studies conducted over the last 20 years, the incidence of SCD among African Americans has consistently been twice as high as that of white Americans, about 3.5 times higher than that of Asian Americans, and about five times higher than that of Hispanic Americans [44]. A higher incidence of myocardial infarction or death was also associated with Asian race [45]. Using a prospective, population-based cohort study, the risk of SCA was evaluated among Hispanics and Asian Americans. The incidence of SCA was similar between white and Hispanic Americans when adjusted for age, but was significantly lower in Asian Americans compared to white Americans by approximately 32% [46]. In sum, the existing literature suggests that further research is necessary to predict the risk of SCD in populations other than white. SCD risk can be determined by differences in biological, secular, and social factors in relation to race with further research in other regions of the world.

There is a direct correlation between the overall sample size and the number of participants with the outcome in prediction model studies. In model development studies, sample size has traditionally been determined by the number of events per variable (EPV). EPVs of at least 10–20 have been widely adopted as a criterion for minimizing overfitting [47, 48]. The incidence of SCD is relatively low in the general population. It is therefore necessary to develop and validate the model using a larger sample size. For the development of the models, the sample size of reviewed studies was 905–20,177, and the number of SCD cases was 13–706, which mostly met the requirements. This is because a larger sample size results in more precise results-that is, smaller standard errors and narrower confidence intervals-as in all medical research.

Currently, predictive models for SCD risk in the community are based on Cox proportional hazards models, which are traditional approaches. According to the C statistic for the final model, excellent discrimination was demonstrated. However, there is an alternative to traditional methods in the form of machine learning-a subfield of artificial intelligence that employs data-driven computational modeling to identify complex patterns in data. Researchers are increasingly using machine learning models to predict SCD risk among patients with tetralogy of Fallot, hypertrophic cardiomyopathy, and Brugada syndrome [49–51]. In genetic status analysis, ECG or image analysis to predict SCD risk, it appears to suggest machine learning may have some incremental utility in predicting SCD over traditional models as well as being better suited to amalgamate complex multidimensional data sources that confer risk than traditional models alone [52].

In assessing the overall bias using PROBAST, twelve studies were scored high risk of bias. There is a lack of detail in the majority of the studies regarding the analysis, and only five studies validated the model. An overfitted machine learning model fails to succeed when presented with unseen data, largely because its capacity exceeds the available information in the data set. Overfitting is difficult to evaluate in the other ten studies. As a result of this systematic review, it is evident that models for SCD risk prediction require further research in order to improve the quality of evidence reporting in this area [6]. The study sorted out the indicators, which can serve to design policies for public health services for SCD screening.

### Limitations

The present study had some limitations. First, as a large list of predictive variables was analyzed, the probability of false-positive findings increased. Second, a conservative approach to excluding duplicate cohorts led to the exclusion of many database studies (e.g., Atherosclerosis Risk in Communities, ARIC). Third, the number of studies that had been included in the meta-analysis was low. It was not possible to assess the existence of publication bias. Fourth, our study considered RRs equivalent to HRs in the resent meta-analysis. Finally, some of the clinical predictors utilized in the studies may not have been reported fully. Hence, this may introduce bias across the studies included in the meta-analysis.

### Conclusion

This systematic review and meta-analysis show that risk prediction model may be useful as an adjunct for risk stratification strategies for SCD in the general population. The most relevant studies are limited to European-American regions, and have high bias risks. Predicting the risk of SCD is an important and exciting area. Further studies among people except for white participants and more accessible factors are necessary to explore this area.

## Data Availability

The data underlying this article are available in the article and in its online Supplementary Material.
