# Supplementary material for: Risk Prediction for Sudden Cardiac Death in the General Population: A Systematic Review and Meta-Analysis
Source: Int J Public Health. 2024 Mar 20;69:1606913. doi: 10.3389/ijph.2024.1606913 (PMC10988292; doi:10.3389/ijph.2024.1606913)
Supplement: Supplementary file 1 [file DataSheet1.ZIP › Additional files/Appendix I.docx]

## Literature search strategy

### Pubmed & Medline

(((Death, Sudden, Cardiac[MeSH Terms]) OR ("sudden heart death*"[Title/Abstract]) OR ("sudden heart arrest*"[Title/Abstract]) OR ("sudden cardiac death*"[Title/Abstract]) OR ("sudden cardiac arrest*"[Title/Abstract]) OR (SCD[Title/Abstract]) NOT ((sickle cell disease[Title/Abstract]) OR (subjective cognitive decline[Title/Abstract]))) OR ("sudden heart event*"[Title/Abstract]) OR ("unexpected heart event*"[Title/Abstract]) OR ("sudden cardiac event*"[Title/Abstract]) OR ("unexpected cardiac event*"[Title/Abstract]) OR (Ventricular Fibrillation[MeSH Terms]) OR (Tachycardia, Ventricular[MeSH Terms]) OR ("ventricular fibrillation "[Title/Abstract]) OR ("ventricular tachycard*"[Title/Abstract]) OR ("ventricular tachyarrhythmi*"[Title/Abstract])) AND ((Risk [MeSH Terms]) OR (Disease Progression [MeSH Terms]) OR ("Sensitivity and Specificity" [MeSH Terms]) OR (Prognosis [MeSH Terms]) OR (“risk assessment*” [Title/Abstract]) OR (“risk scor*”[Title/Abstract]) OR (“risk function*” [Title/Abstract]) OR (“risk equation*”[Title/Abstract]) OR (“risk chart*”[Title/Abstract]) OR (“risk tool*”[Title/Abstract]) OR (“risk assessor*”[Title/Abstract]) OR (“risk appraisal*”[Title/Abstract]) OR (“risk calculation*”[Title/Abstract]) OR (“risk calculator*”[Title/Abstract]) OR (“risk factor*”[Title/Abstract]) OR (“risk table*”[Title/Abstract]) OR (“risk threshold*”[Title/Abstract]) OR (“risk prediction*”[Title/Abstract]) OR (predict* [Title/Abstract]) OR (stratif* [Title/Abstract]) OR (model*[Title/Abstract]) OR (regression* [Title/Abstract]) OR (sensitivity [Title/Abstract]) OR (specificity [Title/Abstract]) OR (likelihood [Title/Abstract]) OR ("ROC curve*" [Title/Abstract]) OR ("AUROC" [Title/Abstract])) NOT (animals[MeSH Terms]) AND (English[Language])

### Embase

('sudden cardiac death'/exp OR 'sudden heart arrest*':ti,ab,kw OR 'sudden heart death*':ti,ab,kw OR 'sudden cardiac arrest*':ti,ab,kw OR 'sudden cardiac death*':ti,ab,kw OR (scd:ti,ab,kw NOT ('sickle cell disease':ti,ab,kw AND 'subjective cognitive decline':ti,ab,kw)) OR 'sudden heart event*':ti,ab,kw OR 'unexpected heart event*':ti,ab,kw OR 'sudden cardiac event*':ti,ab,kw OR 'unexpected cardiac event*':ti,ab,kw OR 'ventricular fibrillation':ti,ab,kw OR 'ventricular tachycard*':ti,ab,kw OR 'ventricular tachyarrhythmi*':ti,ab,kw OR 'heart ventricle tachycardia'/exp OR 'heart ventricle fibrillation'/exp) AND ('risk assessment*':ti,ab,kw OR 'risk scor*':ti,ab,kw OR 'risk function*':ti,ab,kw OR 'risk equation*':ti,ab,kw OR ‘risk chart*’:ti,ab,kw OR ‘risk tool*’:ti,ab,kw OR ‘risk assessor*’:ti,ab,kw OR ‘risk appraisal*’:ti,ab,kw OR ‘risk calculation*’:ti,ab,kw OR ‘risk calculator*’:ti,ab,kw OR ‘risk table*’:ti,ab,kw OR ‘risk threshold*’:ti,ab,kw OR ‘risk prediction*’:ti,ab,kw OR 'predict*':ti,ab,kw OR 'probabilit*':ti,ab,kw OR ' stratif*':ti,ab,kw OR ‘model*':ti,ab,kw OR ‘regression*':ti,ab,kw OR ‘sensitivity':ti,ab,kw OR ‘specificity':ti,ab,kw OR ‘likelihood':ti,ab,kw OR ‘ROC curve*':ti,ab,kw OR ‘AUROC':ti,ab,kw OR ‘cardiometabolic risk’/exp OR ‘cardiovascular risk’/exp OR ‘genetic risk’/exp OR high risk patient/exp OR ‘intermediate risk patient’/exp OR ‘intermediate risk population’/exp OR ‘low risk patient’/exp OR ‘low risk population’/exp OR ‘mortality risk’/exp OR ‘patient risk’/exp OR ‘population risk’/exp OR ‘recurrence risk’/exp OR ‘risk assessment’/exp OR ‘risk behavior’/exp OR ‘risk evaluation and mitigation strategy’/exp OR ‘risk factor’/exp OR ‘risk management’/exp OR risk perception/exp OR 'disease exacerbation '/exp OR 'general condition deterioration'/exp OR 'llness trajectory'/exp OR 'sensitivity and specificity'/exp OR 'maximum likelihood method'/exp OR 'area under the curve'/exp) NOT ('rat':ti,ab,kw OR 'rats':ti,ab,kw OR ‘mouse':ti,ab,kw OR 'mice':ti,ab,kw OR 'swine':ti,ab,kw OR ' porcine ':ti,ab,kw OR ' murine ':ti,ab,kw OR ' sheep ':ti,ab,kw OR ' lambs ':ti,ab,kw OR ‘pigs':ti,ab,kw OR ' piglets ':ti,ab,kw OR ' rabbit':ti,ab,kw OR ' rabbits':ti,ab,kw OR ‘cat’:ti,ab,kw OR ‘cats’:ti,ab,kw OR ‘dog’:ti,ab,kw OR ‘dogs’:ti,ab,kw OR ‘cattle’:ti,ab,kw OR ‘bovine’:ti,ab,kw OR ‘monkey’:ti,ab,kw OR ‘monkeys’:ti,ab,kw OR ‘trout’:ti,ab,kw OR ‘marmoset’:ti,ab,kw OR ' animal experiment '/exp) AND english:la

### Cochrane library

(MeSH descriptor: [Death, Sudden, Cardiac] explode all trees OR (“sudden cardiac death*”):ti,ab,kw OR (“sudden heart arrest*”):ti,ab,kw OR (“sudden heart death*”):ti,ab,kw OR (“sudden cardiac arrest*”):ti,ab,kw OR (“sudden cardiac death*”):ti,ab,kw OR ((scd):ti,ab,kw NOT ((“sickle cell disease”):ti,ab,kw OR (“subjective cognitive decline”):ti,ab,kw)) OR (“sudden heart event*”):ti,ab,kw OR (“unexpected heart event*”):ti,ab,kw OR (“sudden cardiac event*”):ti,ab,kw OR (“unexpected cardiac event*”):ti,ab,kw OR (“ventricular fibrillation”):ti,ab,kw OR (“ventricular tachycard*”):ti,ab,kw OR (“ventricular tachyarrhythmi*”):ti,ab,kw OR MeSH descriptor: [Tachycardia, Ventricular] explode all trees OR MeSH descriptor: [Ventricular Fibrillation] explode all trees) AND (“risk assessment*”):ti,ab,kw OR (“risk scor*”):ti,ab,kw OR (“risk function*”):ti,ab,kw OR (“risk equation*”):ti,ab,kw OR (“risk chart*”):ti,ab,kw OR (“risk tool*”):ti,ab,kw OR (“risk assessor*”):ti,ab,kw OR (“risk appraisal*”):ti,ab,kw OR (“risk calculation*”):ti,ab,kw OR (“risk calculator*”):ti,ab,kw OR (“risk table*”):ti,ab,kw OR (“risk threshold*”):ti,ab,kw OR (“risk prediction*”):ti,ab,kw OR (predict*):ti,ab,kw OR (stratif* ):ti,ab,kw OR (model*):ti,ab,kw OR (regression*):ti,ab,kw OR (sensitivity):ti,ab,kw OR (specificity):ti,ab,kw OR (likelihood ):ti,ab,kw OR ("ROC curve*"):ti,ab,kw OR ("AUROC"):ti,ab,kw OR (MeSH descriptor: [Risk] explode all trees OR (MeSH descriptor: [Disease Progression] explode all trees OR (MeSH descriptor: [Sensitivity and Specificity] explode all trees OR(MeSH descriptor: [Prognosis] explode all trees NOT (MeSH descriptor: [animals] explode all trees

### Web of Science

(TI=(“risk assessment*” OR “risk scor*” OR “risk function*” OR “risk equation*” OR “risk chart*” OR “risk tool*” OR “risk assessor*” OR “risk appraisal*” OR “risk calculation*” OR “risk calculator*” OR “risk table*” OR “risk threshold*” OR “risk prediction*” OR Stratif* OR Model* OR Regression* OR Sensitivity* OR Specificity* OR Likelihood* OR (ROC curve) OR Auroc)) AND (TI=("sudden cardiac death*" OR "sudden cardiac arrest*" OR "sudden heart death*" OR "sudden heart arrest*" OR (SCD NOT ("sickle cell disease" OR "subjective cognitive decline")) OR “sudden heart event*" OR "unexpected heart event*" OR "sudden cardiac event*" OR "unexpected cardiac event*" OR "ventricular fibrillation" OR "ventricular tachycard*" OR "ventricular tachyarrhythmi*”))

### CINAHL

(MW death, sudden, cardiac OR AB "sudden heart death*" OR AB "sudden heart arrest*" OR AB "sudden cardiac death*" OR AB "sudden cardiac arrest*" OR AB "sudden heart event*" OR AB "unexpected heart event*" OR AB "sudden cardiac event*" OR AB "unexpected cardiac event*" OR AB "ventricular fibrillation " OR AB "ventricular tachycard*" OR AB "ventricular tachyarrhythmi*" OR MW ventricular fibrillation OR MW Tachycardia, Ventricular OR (AB SCD NOT AB sickle cell disease NOT AB subjective cognitive decline)) AND (AB “risk assessment*” OR AB “risk scor*” OR AB “risk function*” OR AB “risk equation*” OR AB “risk chart*” OR AB “risk tool*” OR AB “risk assessor*” OR AB “risk appraisal*” OR AB “risk calculation*” OR AB “risk calculator*” OR AB “risk factor*” OR AB “risk table*” OR “risk threshold*” OR “risk prediction*” OR predict* OR stratif* OR model* OR regression* OR sensitivity OR specificity OR likelihood OR ROC curve* OR AUROC OR MW Risk OR MW Disease Progression OR MW ( Sensitivity and Specificity ) OR MW Prognosis) NOT (SU animals)

### OpenGrey

("sudden cardiac death" OR "sudden cardiac arrest" OR "sudden heart death" OR "sudden heart arrest" OR SCD OR "ventricular tachycardia" OR "ventricular tachyarrhythmia" OR "ventricular fibrillation") AND (risk* OR probabilit* OR predict* OR likelihood* OR stratif* OR regression OR hazard* OR mortalit* OR sensitivity* OR specificity OR AUROC OR ROC)

## Inclusion Criteria

- That developed a prediction model.
- To predict SCD risk.
- The study was conducted among the general population (aged 18 and older).

## Exclusion Criteria

- The outcomes of the study were not ventricular fibrillation, ventricular tachycardia, SCA and SCD.
- Type of study: conference proceedings, editorials, reviews, meta-analysis, protocol and short reports.
- Imminent prediction of SCD risk (<1 day).
- That did not develop a prediction model.
- Only genetic status analysis or image analysis.
- The study population was not the general population.

**Meta-analyses were conducted only when the identified predictors were statistically (i.e. type of effect estimates) and clinically homogeneous (e.g. similar reference groups for categorical variables). Meta-Analysis Exclusion Criteria:**

- Studies missing statistical information required for a forest plot analysis.
- When duplicate cohorts are identified using the criteria below, the study with the largest sample size is included in the meta-analysis: (i) A study cohort taken from the same registry as another study cohort. (ii) The same outcome(s) were analyzed. (iii) Overlap between study periods.
