# Supplementary material for: Risk Prediction for Sudden Cardiac Death in the General Population: A Systematic Review and Meta-Analysis
Source: Int J Public Health. 2024 Mar 20;69:1606913. doi: 10.3389/ijph.2024.1606913 (PMC10988292; doi:10.3389/ijph.2024.1606913)
Supplement: Supplementary file 1 [file DataSheet1.ZIP › Additional files/Captain and label of Figure S1-S2 and Table S1.docx]

Figure S1 Prediction model Risk of Bias Assessment Tool for the fifteen studies meeting inclusion criteria (China. 2024)

Notes: A, Prediction model of overall bias using PROBAST; B, Prediction model of applicability using PROBAST

Figure S2 Predictors included in models predicting sudden cardiac death risk in the general population (China. 2024)

Notes: LV indicates Left ventricular; LVH indicates left ventricular hypertrophy; IVST indicates interventricular septal thickness; PWT indicates posterior wall thickness; RWT indicates relative wall thickness; LVIDd indicates LV; LVIDs indicates internal dimensions in end diastole and systole.

Table S1 Definition of sudden cardiac death of all studies (China. 2024)
