# Supplementary material for: Risk Prediction for Sudden Cardiac Death in the General Population: A Systematic Review and Meta-Analysis
Source: Int J Public Health. 2024 Mar 20;69:1606913. doi: 10.3389/ijph.2024.1606913 (PMC10988292; doi:10.3389/ijph.2024.1606913)
Supplement: Supplementary file 1 [file DataSheet1.ZIP › Additional files/Table S1.docx]

**Table S1** **Definition of sudden cardiac death of all studies (China. 2024)**

| **First author** | **Publication year** | **Definition of SCD** |
| --- | --- | --- |
| Sudhir Kurl | 2012 | A death was defined as SCD when it occurred either within 1 hour after the onset of an abrupt change in symptoms or within 24 hours after onset of symptoms when autopsy data did not reveal a noncardiac cause of sudden death or after successful resuscitation from ventricular tachycardia and/or ventricular fibrillation. The deaths due to aortic aneurysm rupture, cardiac rupture or tamponade, pulmonary embolism, cancer, or other noncardiac comorbidities were not included as SCD. Diagnostic classification of events was based on symptoms, ECG findings, cardiac enzyme elevations, autopsy findings (80% of SCDs), and history of CHD together with clinical and ECG findings. |
| Laukkanen Jari A | 2014 | A death was classified as SCD when it occurred within 24 hours of the onset of symptoms, including nonwitnessed cases when clinical and autopsy findings did not reveal a noncardiac cause of sudden death. |
| Sudhir Kurl | 2015 | A death was defined as SCD when it occurred either within 1 h after the onset of an abrupt change in symptoms or within 24 h after onset of symptoms when autopsy data did not reveal a non-cardiac cause of sudden death or after successful resuscitation from ventricular tachycardia and/or ventricular fibrillation. |
| Jonathan W. Waks | 2016 | SCD was defined as a sudden pulseless condition presumed due to a ventricular tachyarrhythmia in a previously stable individual without evidence of a non-cardiac cause of cardiac arrest. |
| Rajat Deo | 2016 | SCD was defined similarly in both ARIC and CHS: a sudden pulseless condition from a cardiac origin in a previously stable individual occurring out of the hospital or in the emergency department. For unwitnessed deaths, the participant must have been seen within 24 hours of the arrest in a stable condition and without evidence of a noncardiac cause of cardiac arrest. |
| Suma H. Konety | 2016 | A sudden pulseless condition presumed to be due to a ventricular tachyarrhythmia in a previously stable individual without evidence of a noncardiac cause of cardiac arrest. |
| Maartje N. Niemeijer | 2016 | A natural death due to cardiac causes, heralded by abrupt loss of consciousness within 1 hour from onset of acute symptoms; preexisting heart disease may have been known to be present, but the time and mode of death are unexpected. |
| Takeki Suzuki | 2016 | A sudden pulseless condition presumed to be due to a ventricular tachyarrhythmia, a separate group of physicians classified definite and possible fatal CHD cases into definite sudden arrhythmic death, possible sudden arrhythmic death, not sudden arrhythmic death, or unclassifiable. Definite and possible sudden arrhythmic deaths composed SCD outcome for this study. |
| Aapo L. Aro | 2017 | SCD was defined as sudden, unexpected loss of the pulse due to a cardiac etiology; and if the death was unwitnessed, subjects were required to have been seen in their usual state of health in the previous 24 hours. Patients with chronic terminal illnesses, end-stage heart failure or with any identifiable non-cardiac causes of death such as pulmonary embolism, aortic dissection, stroke, trauma, drug overdose or intoxication were excluded during the adjudication process. |
| Brittany M. Bogle | 2018 | In ARIC, deaths were reviewed using coroner records, death certificates, and through contacting kin or primary physician. Sudden cardiac death was defined as death resulting from fatal myocardial infarction or definite or possible fatal coronary heart disease where the time between symptom onset and death is less than one hour or where the time between hospital admission and death is less than one hour.  In Framingham, sudden cardiac death was defined as death resulting from coronary heart disease (definite myocardial infarction, coronary insufficiency, or angina pectoris) within one hour of symptom onset with no other probable cause of death suggested from the medical record and interview of relatives. Suspected sudden cardiac death events were adjudicated by a panel of three trained physicians who applied criteria for sudden cardiac death. |
| Arttu holkeri  No data are available. | 2019 | Sudden cardiac death, defined as death of cardiac origin that occurred unexpectedly within 1 hour of the onset of new symptoms or a death that was unwitnessed and unexpected, unless a specific noncardiac cause of death was confirmed. |
| Leonardo Tamariz | 2019 | All cause mortality. |
| Yun-Jiu Cheng  All data relevant to the study are included in the  article or uploaded as supplemental information. All data relevant to the study are  included in the article or uploaded as supplementary information. | 2021 | SCD was defined as an unexpected and sudden pulseless situation of presumed underlying cardiac origin because of the absence of conditions clearly unrelated to cardiac arrhythmias. Study participants were not classified as SCD if there was evidence of an acute non- cardiac morbidity that could account for the death and conditions clearly unrelated to cardiac arrhythmias, such as car accident, drug overdose, cancer, massive blood loss, pneumonitis, pulmonary embolism, acute respiratory failure, stroke, aortic aneurysm rupture and myocardial rupture after myocardial infarction. |
| Anna C. van der Burgh  The datasets analysed during the current study are not publicly available due to legal and ethical restraints. Data are available from the corresponding author on reasonable request | 2022 | a natural death due to cardiac causes, heralded by abrupt loss of  consciousness within 1 h from onset of acute symptoms; preexisting heart disease may have been known to be present, but  the time and mode of death are unexpected. |
| Yun-Yu Chen  The deidentified participant data will not be shared. | 2023 | SCD was defined as a sudden, unexpected, non-traumatic loss of heart function and vital signs without preceding complaints or illness, or occurring within 1h of the onset of complaints. |
