# Supplementary figures and images for: Risk Prediction for Sudden Cardiac Death in the General Population: A Systematic Review and Meta-Analysis
Source: Int J Public Health. 2024 Mar 20;69:1606913. doi: 10.3389/ijph.2024.1606913 (PMC10988292; doi:10.3389/ijph.2024.1606913)

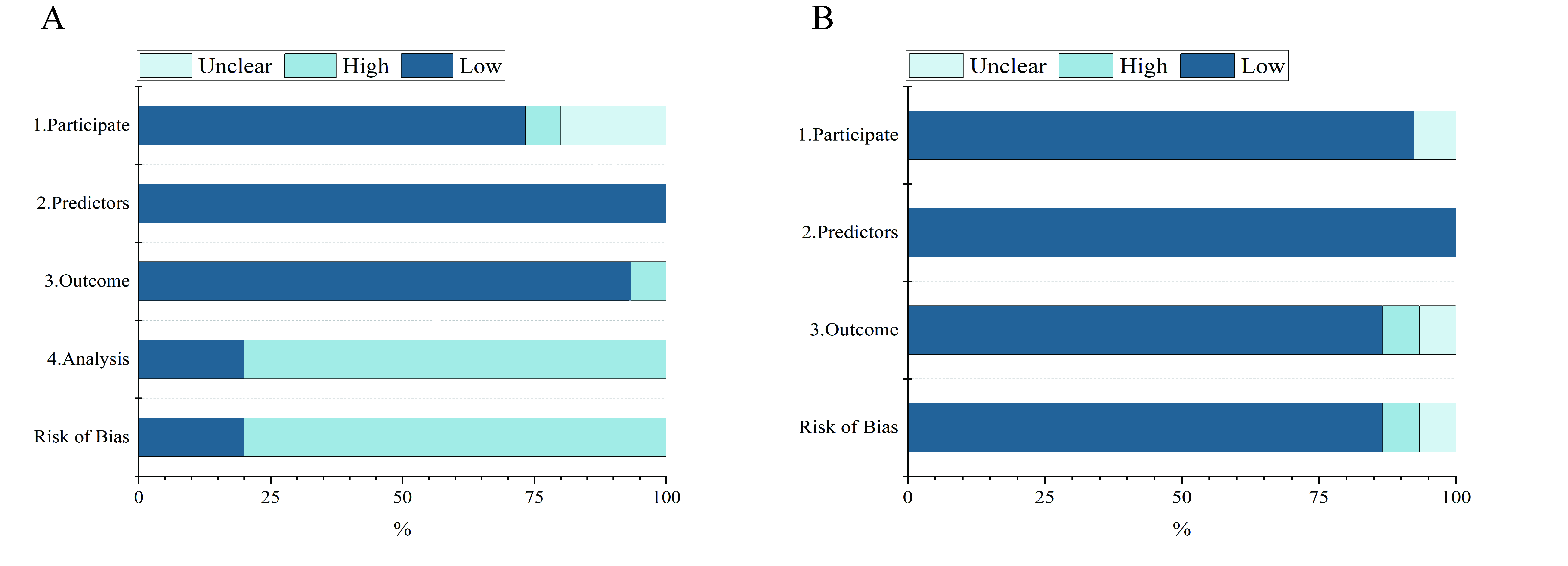

Supplement: Supplementary file 1 [file DataSheet1.ZIP › Additional files/Figure S1.tif]

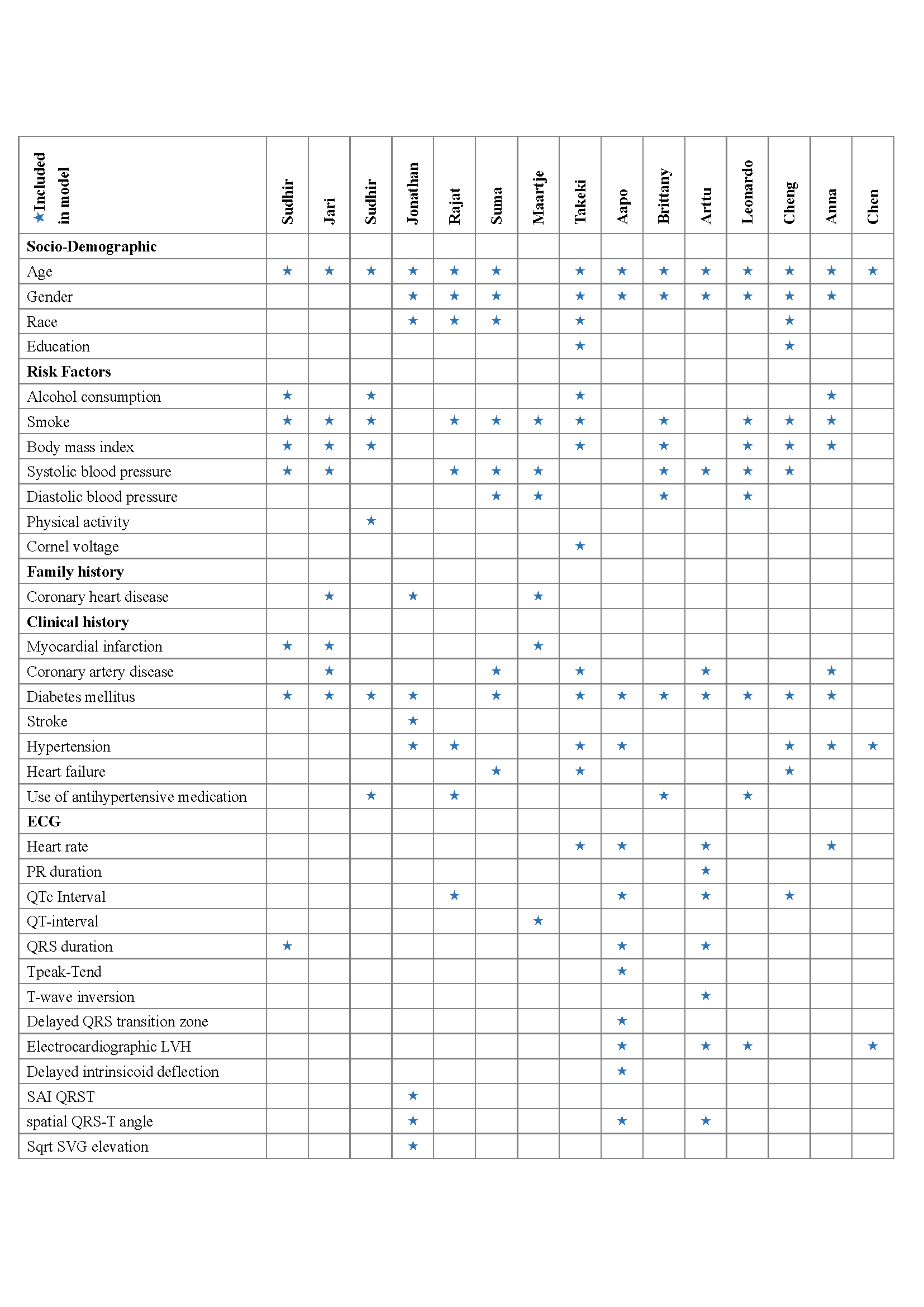

Supplement: Supplementary file 1 [file DataSheet1.ZIP › Additional files/Figure S2-1.tiff]

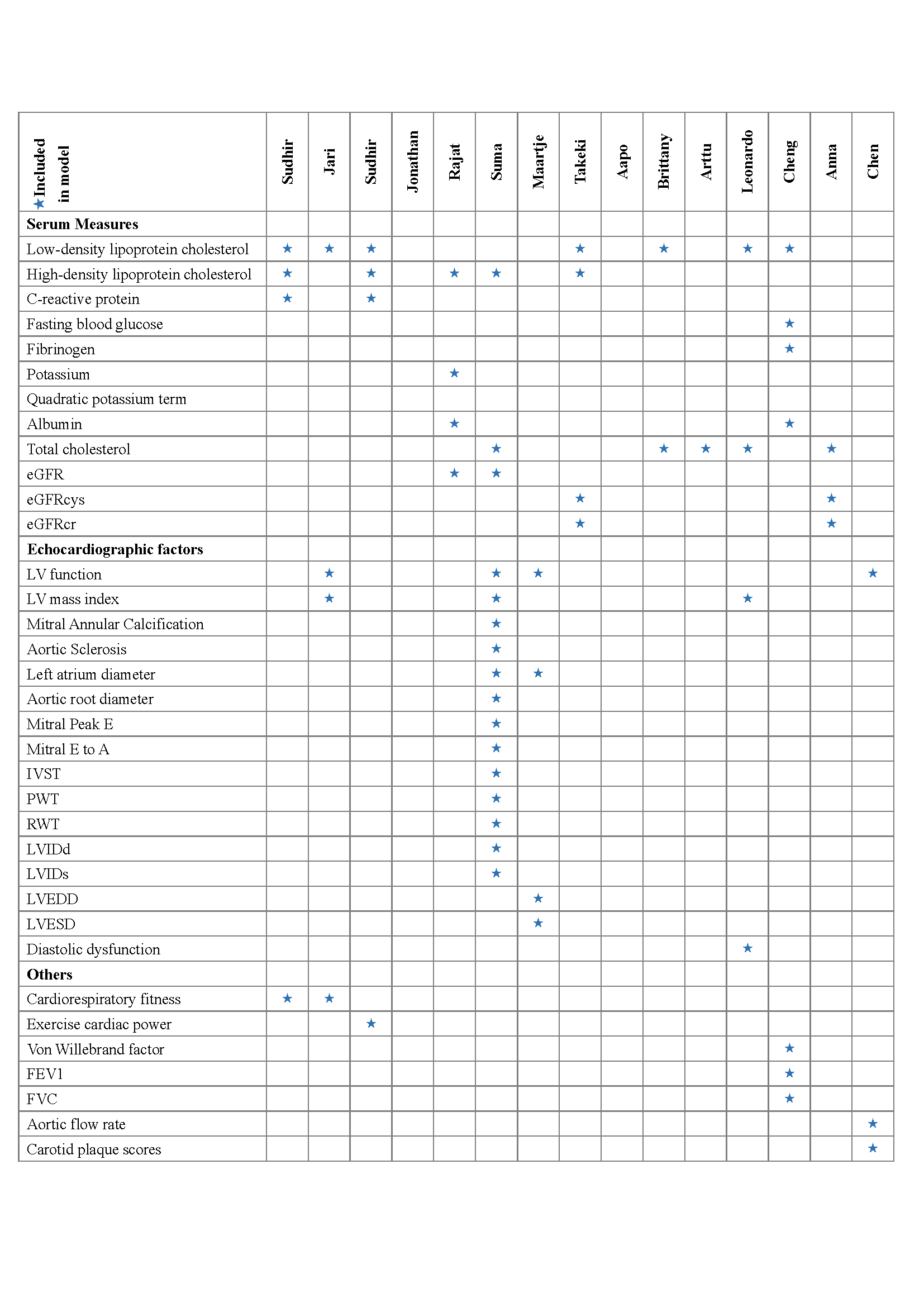

Supplement: Supplementary file 1 [file DataSheet1.ZIP › Additional files/Figure S2-2.tiff]
